# BER Reduction and Capacity Enhancement with Novel Guard Sequence Selection for Multi-Carrier Communication

**DOI:** 10.3390/s23010217

**Published:** 2022-12-25

**Authors:** Oluwaseun Ologun, Shaochuan Wu, Raza Ali Shah, Sohaib Bin Altaf Khattak, Moustafa M. Nasralla

**Affiliations:** 1 Communications Research Center, School of Electronics and Information Engineering, Harbin Institute of Technology, Harbin 150001, China; 2 Department of Electrical Engineering, HITEC University, Taxila 47080, Pakistan; 3 Smart Systems Engineering Lab, College of Engineering, Prince Sultan University, Riyadh 11586, Saudi Arabia

**Keywords:** guard intervals, OFDM, known symbol padding, cyclic prefix, zero padding

## Abstract

Orthogonal frequency division multiplexing (OFDM) is an efficient multicarrier scheme that uses different types of guard intervals such as cyclic prefix (CP) and known symbol padding (KSP) (zero padding (ZP), unique word (UW), etc.) in block formation. Among these guard intervals, CP varies for each block, while other guard intervals remain fixed from block to block. These guard intervals efficiently perform channel estimation, synchronization and remove inter-block interference (IBI); nevertheless, none of the existing schemes develop any relationship between the guard interval (sequence) and the data symbols on different subcarriers of the OFDM block. We present a new idea of selecting the guard interval based on the data symbols of a subset of subcarriers in the block and exploit the high auto-correlation of the selected guard sequence to improve the bit error rate (BER) performance of the system. The results based on a fair comparison show that our enhanced orthogonal frequency division multiplexing (eOFDM) scheme inherits significant improvements in BER and the capacity of a multicarrier system as compared to the existing techniques.

## 1. Introduction

Bandwidth maximization, lower complexity, and reliability are the key attributes that characterize orthogonal frequency division multiplexing (OFDM) as one of the most promising multicarrier transmission schemes since the deployment of fourth generation (4G) services. The apparent importance of OFDM is also evident in the adoption of OFDM and OFDM-based multicarrier schemes for the efficient implementation of the current fifth generation (5G) networks [1] and its supporting technologies [2,3,4,5]. In addition, recent research geared towards the standardization of sixth generation (6G) and beyond networks suggests that OFDM and its optimized modulation variants will not be neglected in the actualization of a 6G connected world [6,7]. Therefore, there is no gainsaying the number of immeasurable opportunities that abound if OFDM and its variants are leveraged by current and future wireless communications.

OFDM variants are broadly classified into cyclic prefix (CP) OFDM, known symbol padding (KSP) OFDM [8,9,10,11], zero padding (ZP) OFDM [12,13], and unique word (UW) OFDM [14,15]. They all have been the focus of investigation among researchers for the last couple of decades. CP-OFDM has been adopted as standard for digital video broadcasting (DVB) in Europe, while KSP-OFDM, also known as time domain synchronous (TDS) OFDM, has governed the Chinese DVB system [16,17,18]. The major difference in these alternatives is the structure of the OFDM block. CP-OFDM uses cyclic prefix as the guard interval (GI) (sequence), which varies from block to block and combats inter-block interference (IBI), while ZP-OFDM uses zeros for this purpose. CP-OFDM is considered to be a promising scheme for fast-fading underwater channels, 5G new radio [19,20], and short packet communications, wherein a prefix and a suffix is used as GI in CP-OFDM [21]. The KSP/TDS-OFDM system uses the PN sequence as a guard interval, which has multi-fold advantages. Apart from IBI removal, it is also suitable for channel estimation and block synchronization due to its high self-correlation properties [22,23,24,25]. UW-OFDM is also considered to be superior to CP-OFDM based on its lower bit error rate (BER) performance and lower out-of-band radiations, specifically in the frequency-selective Rayleigh fading channel [26]. Although both the UW-OFDM and KSP-OFDM systems use the known PN sequence, the former is a frequency domain method. The speedy time domain synchronization and channel estimation make KSP-OFDM superior to UW-OFDM.

The usage of their guard intervals is limited by all of the aforementioned variants of multicarrier schemes; for instance, although each OFDM block’s cyclic prefix in CP-OFDM is unique, the receiver discards it to prevent inter-block interference (IBI), which severely restricts the usage of a variable CP in each block. ZP-OFDM uses zero padding for this purpose and performs identically to CP-OFDM [26]. UW-OFDM [27] and KSP-OFDM use a fixed PN sequence and have some additional benefits of synchronization and channel estimation along with IBI removal. Some other techniques, such as subcarrier number modulation [28], filter bank (FB) OFDM-based index modulation (IM) and hybrid number and index modulation [29,30] schemes, are also reported to improve the BER performance; nevertheless, these schemes assume the CP-OFDM block structure and exploit the number/index of active subcarriers in the block. In contrast to these schemes, our proposed enhanced orthogonal frequency division multiplexing (eOFDM) employs variable guard sequence, which does not require the insertion of pilot subcarriers in each block as in CP-OFDM but still provides scattered data subcarriers in the block for channel estimation. It should be noted that if the number/index of our proposed scheme is also exploited, the gain in BER or throughput as reported by [28,29,30] will be an added advantage.

As far as we know, none of the existing OFDM variants offer a connection between the guard sequence and the data symbols of different subcarriers within the OFDM block. Using a distinct PN sequence from a predefined list of possible sequences as a guard interval for each OFDM block, a novel method of identifying a subset of data symbols using the guard interval is provided. This sequence is constant for one OFDM block but changes based on the subset of data symbols in each block’s data part. The contribution of our scheme lies in the fact that unlike TDS-OFDM, the PN sequence from block to block is not fixed.

In the proposed scheme, the PN sequence is selected from a finite set of available sequences, and the selection is linked to a subset of data symbols, hence, providing the advantages of data symbol detection and additional channel estimation capabilities for various subcarriers. Particularly, our scheme demonstrates an improved bit error rate (BER) in the frequency-selective Rayleigh fading channel. The specific contributions of this paper include:

- A new eOFDM block structure with a variable GI sequence for successive blocks of data, which links the selection of the GI sequence based on the data symbols of scattered data subcarriers;
- A versatile block structure that does not need scattered pilot tones in the OFDM block and still provides the option to use a subset of scattered data symbols as pilot subcarriers;
- The detection of the GI sequence improves the detection performance of a subset of data subcarriers, which results in the improved BER performance of the proposed system.
- A unique and flexible block structure, which provides high spectral efficiency as all the subcarriers of the OFDM block are used as data subcarriers.

Apart from these contributions, a flexible alternate mathematical expression is provided for polynomials of degree *R* with a flexible initial state vector. We highlight the novelty of the proposed scheme in Table 1 by comparing it with the salient features of the fundamental multicarrier OFDM schemes. For a fair comparison of the new scheme with CP-OFDM and TDS-OFDM, the symbol energy, block size, length of the guard sequence and multipath channel impulse response (CIR) for a block are kept the same for all schemes. The CIR varies for successive blocks, as explained in the subsequent section.

The remaining paper is organized as follows. In Section 2, the system model for the proposed eOFDM scheme is presented. The new OFDM block structure is described and compared with the block structure of CP-OFDM and TDS OFDM. Section 3 is dedicated to guard interval analysis. This section describes how the PN sequences are generated and the necessary conditions to be fulfilled by the guard interval sequences. Section 4 presents the performance metrics for fair comparison of CP-OFDM, TDS-OFDM and eOFDM schemes. The numerical results are presented in Section 5, while Section 6 concludes the paper.

## 2. System Model

**Figure 1:**
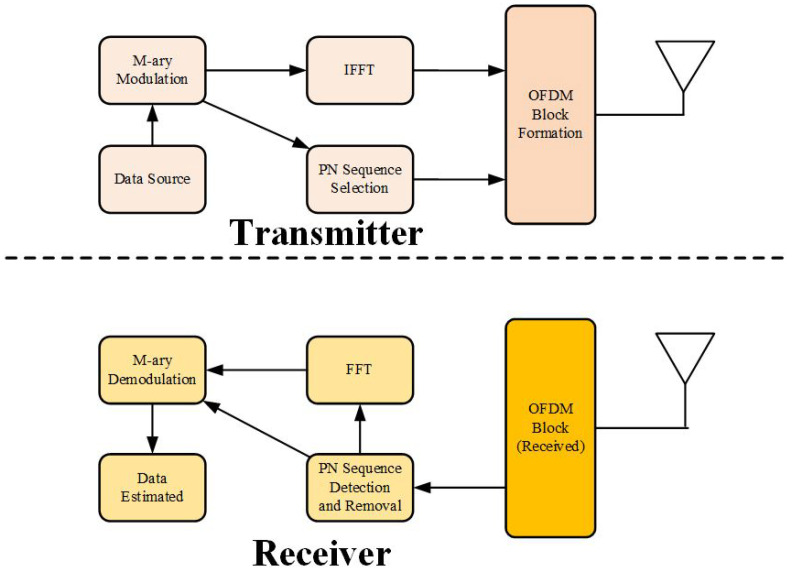
Proposed model for OFDM system.

The block diagram of the proposed eOFDM is shown in Figure 1. The data source generates a binary sequence of 1s and 0s, which are then modulated using an *M*-ary digital modulation scheme. A block of *M*-ary symbols is fed to the IFFT block as well as the PN sequence selection (PNSS) block. The unique PNSS block selects one PN sequence from a group of pre-selected sequences. This selection is based on the subset of *M*-ary data symbols at specified subcarriers, which is described in subsequent text. The selected PN sequence is then used as a guard interval for the eOFDM block, which is transmitted over wireless channel. AT the receiver, the PN sequence detection and removal (PNSDR) process is completed before the FFT operation. It should be noted that a subset of *M*-ary symbols are detected before the FFT operation due to the PNSDR block.

### Proposed eOFDM Block Structure

**Figure 2:**
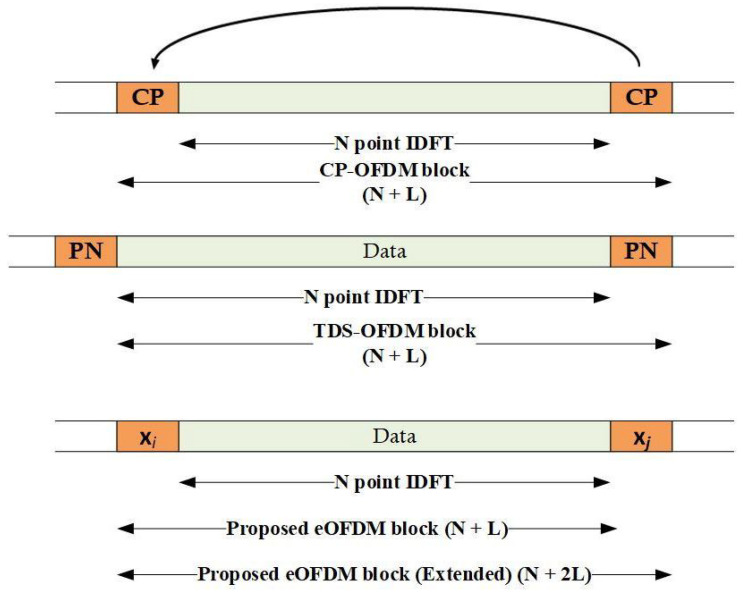
Transmitted blocks of CP-OFDM, TDS-OFDM and eOFDM schemes.

The structure of transmitted blocks for CP-OFDM, TDS-OFDM and the proposed eOFDM systems are compared with the help of the illustration in Figure 2. The set of all OFDM subcarriers in a block are indexed, and a set B=[1,2,…,N] of indices is formed. Set *B* can be split into *G* groups. There are J=N/G subcarriers in each set $B_{g}\subset B,g=1,2,\ldots ,G$. The random source, generating uniformly distributed sequence of 1s and 0s as input, are grouped and mapped to complex valued set of symbols $s={\left[s_{1},s_{2},\ldots ,s_{N}\right]}^{T}$ chosen from an *M*-ary digital modulation scheme, where ${\left(.\right)}^{T}$ represents the transposition operations. Although, in general, any M≥2 can be used for data symbols, we select M=2 for the brevity and simplicity of the new idea. Each subset $B_{g}$ of the subcarriers are used to transmit *J* complex valued symbols from s. The *N* point IDFT operation of s yields $x={\left[x_{1},x_{2},\ldots ,x_{N}-1\right]}^{T}$, which is a time domain sequence, where the real valued $x_{n}$ can be expressed as [31]

(1) $$x_{n}=\frac{1}{\sqrt{N}}\sum_{k=0}^{N-1}X_{k}^{\prime }e^{j2\pi n\frac{k}{N}},n=0,1,\ldots ,N-1$$

The guard interval is selected from the set of a well-known PN sequence $\{x_{d}\},$$d=1,2,\ldots ,2^{G}$, which exhibits the constant amplitude and zero cross-correlation properties in the time domain [32,33,34,35]. The unique feature of eOFDM is the selection of $x_{d}$, which is based on $s_{i}$s carried by the subcarriers with indices from $B_{o}^{\prime }\subset B$, where $B_{o}^{\prime }=\left\{o_{1},o_{2},\ldots ,o_{G}\right\}$ and $o_{i}\in \left\{1,2,\ldots ,J\right\}$ for each $B_{g}$. It should be noted that $B_{o}^{\prime }$ is formed from the available set of *N* subcarriers with only one subcarrier from each $B_{g}$. To combat the effect of multipath channels with *L* significant paths, a sequence $x_{d}=\left[x_{d_{1}},x_{d_{2}},\ldots ,x_{d_{m}}\right]$ is appended at the beginning of the block. It is emphasized that in general m≥L. The block $b={\left[x_{d}\;x\right]}^{T}$ is thus formed for transmission over the channel, which can be seen in Figure 2. The extended block is also shown in Figure 2. It is important to bear in mind that if the channel is modeled as linear convolution, then b resembles the TDS-OFDM block structure; nevertheless, for circular convolution, a necessary condition $x_{i}=x_{j}$ must be fulfilled, where $x_{i},x_{j}\in \left\{x_{d}\right\}$. The multipath channel with time-varying path gains is assumed for signal propagation, which can be expressed as

(2) $$h\left(t,\tau \right)=\sum_{l=1}^{L}\alpha_{l}\left(t\right)\delta \left(\tau -\tau_{l}\left(t\right)\right)$$

where $\alpha_{l}$ and $\tau_{l}\left(t\right)$ are the time-varying amplitude and delay component of path *l*, respectively, while δ(t) is the Dirac delta function. The $\tau_{l}\left(t\right)=\tau_{l}+\Delta_{l}t$ encompasses the effect of the time-varying Doppler scaling factor, $\Delta_{l}$. The multipath CIR with the fixed Doppler scaling factor, Δ, for all paths can be expressed as

(3) $$h\left(t,\tau \right)=\sum_{l=1}^{L}\alpha_{l}\left(t\right)\delta \left(\tau -\tau_{l}+\Delta t\right)=\sum_{l=1}^{L}h_{l}$$

**Figure 3:**
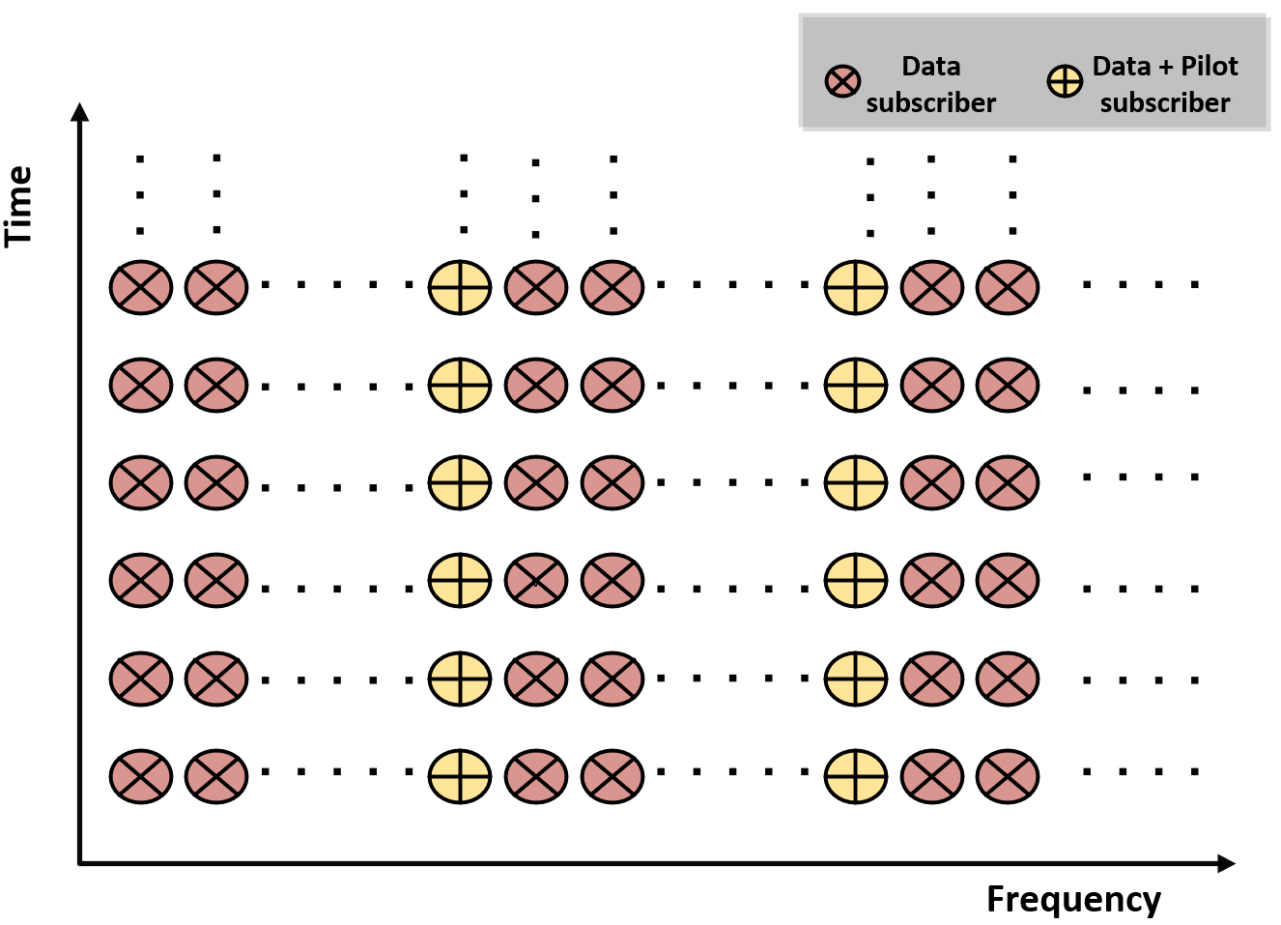
Block structure of eOFDM system.

The multipath channel can be expressed in vector form as $h=[h_{1},h_{2},\ldots ,h_{L}]$. The convolution of the OFDM block $b^{T}$ and h, i.e., $r=b^{T}*x_{d}$, yields a vector of the time domain received sequence $r=[r_{1},r_{2},\ldots ,r_{N+m+L-1}]$. The data part from r can be obtained as $r^{\prime }=\left[r_{m+1},r_{m+2},\ldots ,r_{N+m}\right]$ after the subtraction of $e=h^{T}*x_{d}$ from r. The detection of $x_{d}$, due to zero cross-correlation properties, results in a reduced error probability of *G* symbols, which becomes more significant in highly frequency-selective channels, as can be observed in the subsequent section. The time-frequency view of the eOFDM block is also shown in Figure 3, where the subcarriers with indices from set $B_{o}^{\prime }$ are labeled as data+pilot subcarriers. Each subcarrier with an encircled plus sign is actually a data subcarrier and can also be used as pilot, as the data symbols on these subcarriers are used to select the guard interval sequence of the block. The significance of this time-frequency view of the eOFDM block lies in the fact that although it is similar to the time-frequency view of scattered pilots in the OFDM block structure, there is no pilot insertion in the proposed eOFDM block. All the *N* subcarriers are data subcarriers; a sub-group of these with an encircled plus sign are linked to the selection of a PN sequence at the transmitter. Therefore, the data symbols on these subcarriers are detected before the FFT operation at the receiver as soon as PNSDR detects the PN sequence. It should be noted that these subcarriers inherit additional channel estimation capabilities within the eOFDM block, which is useful for fast and frequency-selective fading channels. Therefore, these are labeled as data+pilot subcarriers.

## 3. Guard Interval

The novelty of the proposed eOFDM scheme is signified by the unique way of linking the guard interval to random user data. This section provides necessary conditions for selecting pre-defined sets of PN sequences for transmission. The set of sequences for guard interval are generated using maximum-length linear shift registers with the length

$$m=2^{L}-1.$$

The generator polynomial, in general, can be expressed as

(4) $$\begin{array}{ccc} P_{j}\left(Z\right) & =Z^{q_{R}}+\ldots +Z^{q_{2}}+Z^{q_{1}}+1, & \;j=1,\ldots ,2^{G} \end{array}$$

where

$$q_{i}=\left\{\begin{array}{cc} i\times b_{i} & ,i=1,2,\ldots ,R-1\\ R & ,i=R \end{array}\right.$$

and $b_{i}\in \left\{0,1\right\}$, while *R* denotes the degree of the polynomial. The initial state vector can be represented as

$$t=[t_{R},t_{R-1},\ldots ,t_{0}]$$

where $t_{i}\in \left\{0,1\right\}$, with the condition that t is a non-zero vector. Let $y_{d}=\left[y_{0},y_{1},\ldots ,y_{m-1}\right]$ be the received PN sequence. Its cross-correlation with each of the sequences $\left\{x_{d}\right\}$ is defined as

(5) $$\begin{array}{ccc} C_{i} & =\sum_{k=0}^{m-1}y_{k+i}x_{d_{k}}, & \;i=1,\ldots ,m \end{array}$$

Furthermore, the cross-correlation $C_{i}$ for the noiseless case is defined as [36]

(6) $$C_{i}=\left\{\begin{array}{cc} m & ,\mathrm{iff}\;y_{d}=x_{j}\\ \leq 0 & ,\mathrm{iff}\;y_{d}\neq x_{j} \end{array}\right.$$

Each of the sequences in $\left\{x_{d}\right\}$ are generated using $P_{j}\left(Z\right),j=1,\ldots ,2^{G},$ which fulfills the necessary condition mentioned in (6). The probability of the correct detection of $y_{d}$ approaches unity at SNR≥−15 dB, as mentioned in [36].

### Generation and Selection of GI

The selection of a variable PN sequence as GI is one of the novel features of eOFDM, which inherits numerous benefits of the flexible proposed block structure; therefore, this section is specifically added to describe how the final subset of PN sequences is formed for the eOFDM transmission scheme. Initially, PN sequences are generated using the generator polynomial according to (4). It should be noted that (4) provides an option to use all possible polynomial equations of degree *R* for generating a very large set of PN sequences. This is achieved by setting either $q_{i}=0,i\neq R$ and/or any $t_{i}=0$. Notably, with these two conditions, (4) becomes the most flexible equation for a polynomial of degree *R*. It allows us to select a polynomial of degree *R* with any initial condition and any polynomial equation. Alternatively, we can say that the initial set of polynomials contains the PN sequences generated from all possible polynomials of degree *R* with all possible initial-state vectors. Hence, as a first step, all the possible PN sequences of degree *R* are generated to form a very large set of PN sequences. This initial large set is then scrutinized according to (6), and most of the PN sequences, which do not fulfill the cross-correlation condition of (6), are dropped. This results in a small subset of PN sequences that fulfill the conditions stated in (6). One of the PN sequence from this subset is then selected for an eOFDM block according to the data on scattered data subcarriers of set $B_{o}^{\prime }$. The data symbols on these scattered data subcarriers are linked to the PN sequence of the eOFDM block, which is used as a GI; hence, the high detection probability of GI improves the detection probability of these scattered data symbols, which results in the improved BER performance of the system. Furthermore, these scattered data symbols can be used as pilot symbols in a way similar to CP-OFDM, which is an additional flexibility to be investigated for channel estimation as a future research problem for an eOFDM block structure.

**Figure 4:**
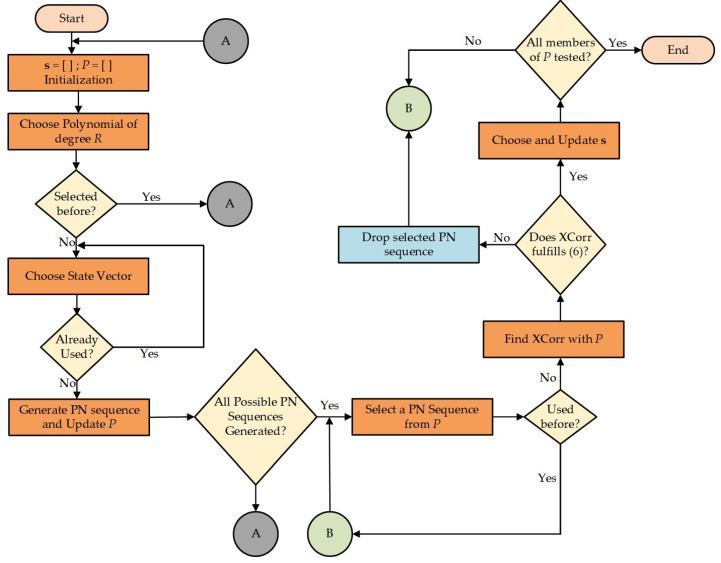
Novel GI sequence generation and selection for eOFDM scheme.

The procedure of generating a final subset of GI sequences is explained with the help of flow chart, as shown in Figure 4. As a first step, two empty sets, *S* and *P*, are initialized, followed by the selection of an unused polynomial of degree *R* and the initial state vector. A set of possible PN sequences is generated, and set *P* is filled. This process is repeated until all the PN sequences of a given degree are generated. Each member of set *P* satisfying the condition stated in (6) is, then, copied to a subset *S*. This subset *S* is used for eOFDM block formation.

## 4. Performance Metrics

This section is dedicated to the analysis of various performance metrics, such as spectral efficiency, energy efficiency, and capacity. The proposed eOFDM scheme is compared based on these parameters with TDS-OFDM and CP-OFDM.

### 4.1. Spectral Efficiency

The spectral efficiency ($\eta_{s}$) of CP-OFDM, TDS-OFDM, and eOFDM systems is compared according to the expression given in [37] as

(7) $$\begin{array}{c} \eta_{s}=\frac{N_{\mathrm{Data}}}{N_{\mathrm{Data}}+N_{\mathrm{Pilot}}}+\frac{N}{N+m}\times 100\% \end{array}$$

where $N_{\mathrm{Data}}$ and $N_{\mathrm{Pilot}}$ represents the number of data subcarriers and the number of pilot subcarriers, respectively, while and $N=N_{\mathrm{Data}}+N_{\mathrm{Pilot}}.$ It should be noted that for eOFDM, $N_{\mathrm{Pilot}}=G.$

### 4.2. Energy Efficiency

The energy efficiency ($\eta_{e}$) of the three OFDM systems is compared according to

(8) $$\begin{array}{c} \eta_{e}=\frac{N_{\mathrm{Data}}}{N_{\mathrm{Data}}+\beta^{2}N_{\mathrm{Pilot}}}+\frac{N}{N+\alpha^{2}m}\times 100\% \end{array}$$

The scalars $\alpha =\sqrt{2}$ and β=4/3 are used for the computation of $\eta_{e}$ for TDS-OFDM, while for CP-OFDM, α=1 is assumed [37].

### 4.3. Capacity Analysis

The capacities of CP-OFDM, TDS-OFDM, and eOFDM are compared based on

(9) $$\begin{array}{c} C=\max_{p(x)}\;I\left(X;Y\right) \end{array}$$

where I(X;Y) represents the mutual information between channel input *X* and channel output *Y* over all input probability distributions of the channel. It should be pointed out that for the given transmission schemes, binary phase shift keying (BPSK) modulation and hard decision decoding (9) can be simplified as

(10) $$\begin{array}{c} C=1-H_{b}\left(\epsilon \right) \end{array}$$

where $H_{b}\left(.\right)$ represents a binary entropy function, and ϵ denotes the probability of the bit error.

## 5. Numerical Results

**Figure 5:**
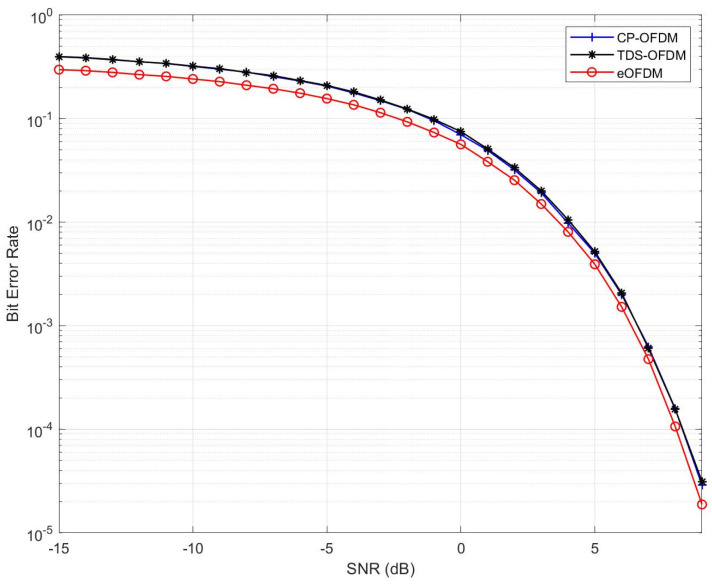
BER comparison of CP-OFDM, TDS-OFDM and eOFDM in AWGN channel.

**Figure 6:**
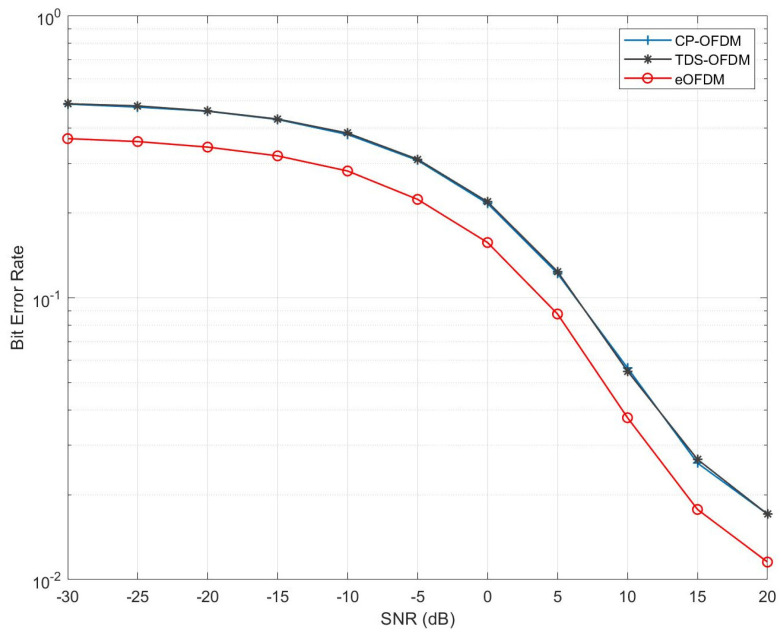
BER comparison of CP-OFDM, TDS-OFDM and eOFDM in multipath Rayleigh fading channel.

This section presents numerical results for the fair comparison of the eOFDM scheme with the CP-OFDM and TDS-OFDM schemes and describes the development of a simulation model. The spectral efficiency, energy efficiency, BER and capacities of the systems were compared. The symbol energy, block size (N), length of the guard sequence PN/CP, and multipath channel impulse response (CIR) for one block remained the same for all schemes. The numerical results for $\eta_{s}$ and $\eta_{e}$ are shown in Table 2. It is worth mentioning that eOFDM is more spectrally efficient than CP-OFDM for $\left(x_{i}=x_{j}\right)$ and equals TDS-OFDM for $\left(x_{i}\neq x_{j}\right)$. Based on the $\eta_{e}$ comparison, it is concluded that eOFDM is as energy efficient as TDS-OFDM for $\left(x_{i}=x_{j}\right)$, while it outperforms both TDS-OFDM and CP-OFDM for $\left(x_{i}\neq x_{j}\right)$. Table 3 provides the simulation parameters for the fair comparison of eOFDM. It is worth mentioning here that the proposed system model did not impose any restrictions on the simulation parameters. Generally, any higher values of these parameters, such as *N*, are supported. These parameter values were merely selected to avoid any unnecessary complexity and brevity of the proposed model. The simulation model was developed according to the proposed system model, as shown in Figure 1. The uniformly distributed binary source represents the data source, which output binary 1s and 0s. This sequence was then modulated using the BPSK modulation scheme. A vector of modulated symbols was formed for the IFFT operation. The PN sequence was selected for insertion as the guard interval. This selection was based on a vector of every *J*-th symbol from $B_{g}$. PN sequence generation and selection is a unique block that was included in the eOFDM system model. A set of PN sequences was formed using a conventional PN sequence generator. Then, a subset, which fulfilled the condition stated in (6), was selected. The PN sequences in this subset were used for insertion in OFDM symbols. Figure 5 compares the BER results obtained for BPSK modulation in an additive white Gaussian noise (AWGN) channel. It can be observed that eOFDM performs better than CP/TDS OFDM for the given range of SNR. This SNR gain is the result of the high probability of detection of $x_{d}$ or *G* data symbols in the block, as mentioned in [36]. The SNR gain for the given BER is more prominent in Figure 6.

**Figure 7:**
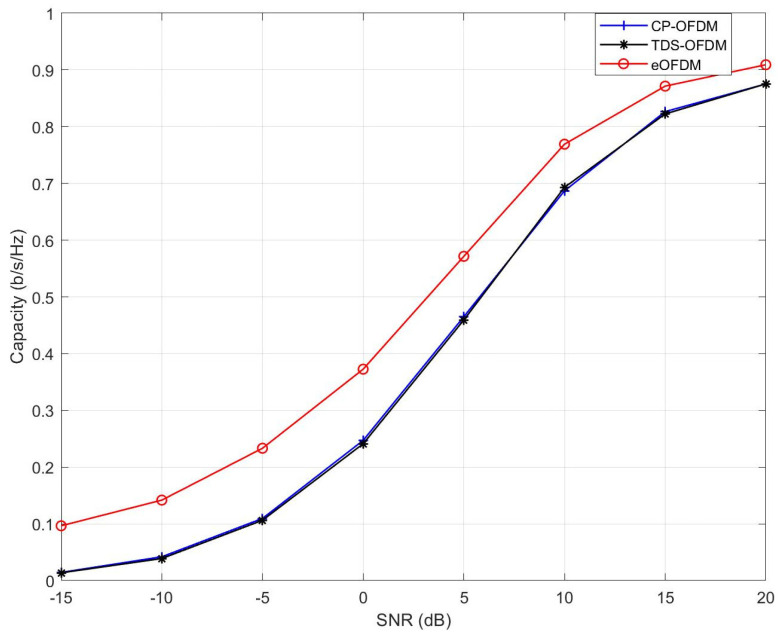
Capacity of CP-OFDM, TDS-OFDM and eOFDM in multipath Rayleigh fading channel.

These results were obtained for the ITU pedestrian B channel model [38]. The variations of channel realization for each OFDM block were considered according to the average path delays (seconds) and path gain (dB) vectors, as mentioned in the Table 3. The channel impulse responses with L=6 were generated for a 50 Hz Doppler frequency $\left(f_{D}\right)$. Notably, the eOFDM scheme shows an approximately 3 dB SNR gain for the BER $10^{-1}$. The SNR gain of eOFDM can be observed for any value of BER, which is high for a high BER and has a decreasing trend for lower BER values. These results validate that, based on a fair comparison, the eOFDM scheme outperforms the CP-OFDM and TDS-OFDM schemes for the given range of SNR in a frequency-selective Rayleigh fading channel. The system capacity of the eOFDM scheme was compared with CP-OFDM and TDS-OFDM, as depicted in Figure 7. These graphs are plotted according to Equation (10). It is worth mentioning that the hard decision decoding and lower modulation order made it feasible to represent system capacity in bits/seconds/Hz (b/s/Hz) for eOFDM, CP-OFDM and TDS-OFDM. It can be observed that the system employing eOFDM has a higher capacity than CP-OFDM and TDS-OFDM for the given range of SNR.

## 6. Conclusions

In this article, we proposed a novel enhanced OFDM (eOFDM) scheme that was established on the linkage of guard interval sequences to user data. We introduced a variable PN sequence (guard sequence), which was selected according to data symbols carried by a subset of subscribers and has the advantage of eliminating the need for pilot symbol insertion while also possesing channel estimation capabilities for different subscribers. Our simulation results, which were based on a fair comparison, showed a reduction in the bit error rate (BER) and an increased capacity when compared to both TDS-OFDM and CP-OFDM. Additionally, our eOFDM showed a better performance in spectral efficiency and energy efficiency in relation to CP-OFDM and demonstrated an improved spectral efficiency and energy efficiency compared to TDS-OFDM depending on the guard interval selection criterion. Thus, our scheme outperforms TDS-OFDM and CP-OFDM based on capacity and BER performance for a wide range of SNR. 

## Figures and Tables

**Table 1:** Comparison of CP-OFDM, TDS-OFDM and eOFDM salient features.

	Guard Interval Significance	Pilot Tones and Flexibility
**CP-OFDM**	Variable CP as GI in each OFDM block is used, which removes ISI	Scattered pilot tones compromises SE; simplified but inflexible block structure
**TDS-OFDM**	Fixed PN sequence as GI in each OFDM block is used, which removes ISI with additional CE capability	No pilot tones, hence no compromise on SE; simplified but inflexible block structure
**eOFDM**	Novel variable PN sequence as GI is used, which removes ISI, improves block BER with additional CE capability	No pilot tones, hence, no compromise on SE, but nevertheless, a subset of data symbols can be used as pilot tones as in CP-OFDM (future extension (FE)) without any compromise on SE; flexible block structure (FE)

**Table 2:** Comparison of $\eta_{s}$ and $\eta_{e}$ for m=N/4.

	CP-OFDM	TDS-OFDM	eOFDM $\left(x_{i}\neq x_{j}\right)$	eOFDM $\left(x_{i}=x_{j}\right)$
$\eta_{s}$	60%	80%	80%	66.67%
$\eta_{e}$	65.23%	66.67%	80%	66.67%

**Table 3:** Simulation parameters for OFDM block and ITU pedestrian B channel model.

Simulation Parameter	Value
Number of channel realizations (h)	10,000
Path delays (s)	[0, 200, 800, 1200, 2300, 3700] 10${}^{-9}$
Average path gains (dB)	[0,−0.9,−4.9,−8,−7.8,−23.9]
Doppler frequency $\left(f_{D}\right)$	50 Hz
Number of OFDM blocks	10,000
IDFT/DFT size (N)	32
Ratio of CP/PN to *N* (v)	1/4
Modulation order (M)	2

## Data Availability

Not applicable.
